# Mortality after acute kidney injury and acute interstitial nephritis in patients prescribed immune checkpoint inhibitor therapy

**DOI:** 10.1136/jitc-2021-004421

**Published:** 2022-03-29

**Authors:** Megan L Baker, Yu Yamamoto, Mark A Perazella, Nazli Dizman, Anushree C Shirali, Navid Hafez, Jason Weinstein, Michael Simonov, Jeffrey M Testani, Harriet M Kluger, Lloyd G Cantley, Chirag R Parikh, F Perry Wilson, Dennis G Moledina

**Affiliations:** 1 Section of Nephrology, Department of Internal Medicine, Yale School of Medicine, New Haven, CT, USA; 2 Clinical and Translational Research Accelerator, Department of Internal Medicine, Yale School of Medicine, New Haven, CT, USA; 3 Department of Internal Medicine, Yale School of Medicine, New Haven, Connecticut, USA; 4 Section of Medical Oncology, Department of Internal Medicine, Yale School of Medicine, New Haven, CT, USA; 5 Section of Cardiovascular Medicine, Yale School of Medicine, New Haven, CT, USA; 6 Division of Nephrology, Johns Hopkins University, Baltimore, MD, USA

**Keywords:** Biostatistics, Immunotherapy, Active, Translational Medical Research

## Abstract

**Background:**

In patients receiving immune checkpoint inhibitor (ICI) therapy, acute kidney injury (AKI) is common, and can occur either from kidney injury unrelated to ICI use or from immune activation resulting in acute interstitial nephritis (AIN). In this study, we test the hypothesis that occurrence of AIN indicates a favorable treatment response to ICI therapy and therefore among patients who develop AKI while on ICI therapy, those with AIN will demonstrate greater survival compared with others with AKI.

**Methods:**

In this observational cohort study, we included participants initiated on ICI therapy between 2013 and 2019. We tested the independent association of AKI and estimated AIN (eAIN) with mortality up to 1 year after therapy initiation as compared with those without AKI using time-varying Cox proportional hazard models controlling for demographics, comorbidities, cancer type, stage, and therapy, and baseline laboratory values. We defined eAIN as those with a predicted probability of AIN >90th percentile derived from a recently validated diagnostic model.

**Results:**

Of 2207 patients initiated on ICIs, 617 (28%) died at 1 year and 549 (25%) developed AKI. AKI was independently associated with higher mortality (adjusted HR, 2.28 (95% CI 1.90 to 2.72)). Those AKI patients with eAIN had more severe AKI as reflected by a higher peak serum creatinine (3.3 (IQR 2.1–6.1) vs 1.4 (1.2–1.9) mg/dL, p<0.001) but exhibited lower mortality than those without eAIN in univariable analysis (HR 0.43 (95% CI 0.21 to 0.89)) and after adjusting for demographics, comorbidities, and cancer type and severity (adjusted HR 0.44 (95% CI 0.21 to 0.93)).

**Conclusion:**

In patients treated with ICI, mortality was higher in those with AKI unrelated to ICI but lower in those where the underlying etiology was AIN. Future studies could evaluate the association of biopsy-proven or biomarker-proven AIN with mortality in those receiving ICI therapy.

## Background

The use of immune checkpoint inhibitors (ICIs) has rapidly increased over the past decade and has significantly improved the survival of patients with various malignancies. ICIs have become standard treatments for various common malignancies such as melanoma, non-small cell lung cancer, microsatellite instability-high tumors, and many others, and are increasingly studied for their potential role in the treatment of other malignancies.^1–3^ These therapies have shown improved survival by months to years compared with other standard-of-care treatments in many clinical settings.^1^

Acute kidney injury (AKI) is a known complication in patients on ICI therapy. AKI incidence ranges from only 2%–3% in clinical trials^4–6^ to up to 17% in real-world analyses.^7–10^ Case series of kidney biopsies in patients experiencing ICI-associated AKI use report that acute interstitial nephritis (AIN) is the histological diagnosis in over 80% of cases.^11 12^ AIN is thought to be an immune-related adverse event (IrAE).^13^ IrAEs are off-target effects of T-cell activation against non-tumor cells resulting in damage to various organs, including the kidney.^12^ However, the true etiological spectrum of ICI-associated AKI also includes acute tubular injury (ATI) and prerenal azotemia. AKI could also be due to coadministered drugs such as chemotherapy and vascular endothelial growth factor-pathway targeting drugs or due to morbidity related to malignancy itself, such as postrenal obstruction from tumor, kidney involvement by tumor, and coagulopathy. Non-AIN causes of AKI may be significantly underrepresented in biopsy series since these cases, when suspected, are usually managed without a biopsy. In fact, a review of cases with ICI-associated AKI without biopsy showed that most of the cases were not attributable to ICI use.^9 10^ Thus, evaluation of ICI-associated AKI involves careful determination of its underlying etiology and may require a kidney biopsy to unequivocally establish the diagnosis.

It is not clear if the occurrence of ICI-associated AKI has any impact on long-term outcomes. Occurrence of AKI could lead to premature discontinuation of life-saving ICI therapy and unnecessary immune suppression if incorrectly diagnosed. AKI from ATI or prerenal azotemia also occurs as a result of the overall disease burden and is associated with higher mortality in other settings. On the other hand, several studies have suggested that the occurrence of dermatologic, endocrinologic, and low-grade IrAEs indicates ICI effectiveness and is associated with improvements in patient outcomes.^1 14–19^ Thus, it is possible that a subset of patients with AKI who have AIN may in fact have better survival. Whether the association of AKI with mortality differs between those who experience AKI due to AIN or other causes is not known. While it is difficult to diagnose AIN without a kidney biopsy,^20–22^ we recently developed and externally validated a statistical model for diagnosis of AIN using data from participants with biopsy-proven AIN and controls.^23^ In this study, we test the association of model predicted AIN with mortality among participants on ICI therapy who develop AKI. We hypothesize that occurrence of AIN indicates a favorable response to ICI therapy; therefore, among patients who develop AKI while on ICI therapy, those predicted to have AIN will demonstrate greater survival compared with others with AKI.

## Methods

### Participants and settings

In this observational cohort study, we included participants in the Yale-New Haven Health System initiated on any one of six ICIs including ipilimumab, nivolumab, pembrolizumab, durvalumab, atezolizumab, or tremelimumab between February 2013 and January 2019, and had at least one serum creatinine value available before drug initiation. We conducted follow-up until July 2019. We excluded participants without any follow-up information or serum creatinine values, or those who died within 15 days after of drug initiation.

### Exposure and outcomes

Our primary outcome was mortality assessed after 15 days and up to 365 days of ICI therapy initiation. A 15-day delay prior to the assessment period was implemented to limit inclusion of AKI events occurring around the time of initiation of ICI therapy which were most likely unrelated to ICI therapy.

Our exposure of interest was the occurrence of AKI and estimated AIN (eAIN) after initiation of ICI therapy. We defined AKI as a 50% increase in serum creatinine from baseline, which corresponds to Kidney Diseases: Improving Global Outcomes (KDIGO) serum creatinine criterion for AKI.^24^ Baseline serum creatinine was defined as the creatinine value obtained immediately prior to ICI initiation.^25^ In a sensitivity analysis, we altered the definition of baseline creatinine to be the mean of all serum creatinine values obtained within 6 months prior to ICI initiation.^9^

We defined eAIN as those who had >90th percentile probability of AIN using a previously published validated diagnostic model for biopsy-proven AIN.^23^ We chose 90th percentile probability as 10% of all cases of biopsied AKI cases had AIN in prior studies.^26 27^ This score was developed and validated in a cohort of participants with biopsy-proven AIN and controls. Its components include serum creatinine at the time of AKI, blood urea nitrogen to creatinine ratio, urine specific gravity, and urine protein (online supplemental figure S1). A higher score indicates greater predicted probability of AIN. In a sensitivity analysis, we used predicted probability of AIN as a continuous exposure. In another sensitivity analysis, we defined eAIN as those with predicted probability of AIN >97th percentile based on the 3% reported cases of AKI directly attributed to ICI use.

10.1136/jitc-2021-004421.supp1 Supplementary data

**Figure 1:**
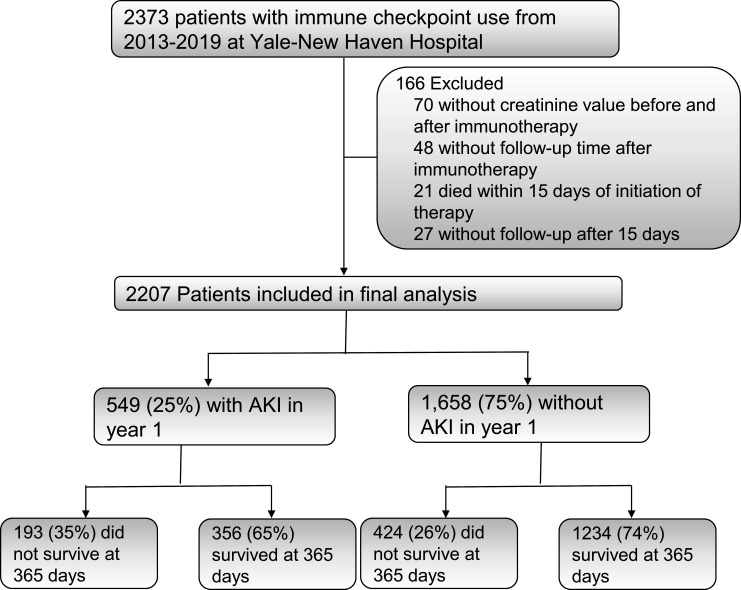
STARD flow diagram. Survival assessed up to 1 year after initiation of immune checkpoint inhibitor therapy. AKI, acute kidney injury.

A board-certified nephrologist (DGM), blinded to model probability, adjudicated 10 patients with predicted probability >90th percentile and 10 patients with predicted probability <10th percentile and provided data on the most likely cause of AKI. We also collected data on most likely etiology of AKI as documented by the treating clinicians through chart review. Finally, we classified AKI into categories proposed in a recent review.^10^

We also evaluated AKI stage and duration. We classified AKI into stage 1 defined as 50%–100% increase in creatinine from baseline and stage 2 or higher as >100% increase in creatinine or requiring dialysis, which correspond to KDIGO AKI stages.^24^ Duration of AKI was defined as the number of days from when a participant first met AKI criterion to when they no longer met this criterion. For this definition, we only included participants with at least one serum creatinine value within 28 days of AKI in addition to the creatinine value used to define AKI. We defined short duration AKI if the duration was ≤7 days and long duration if the duration was >7 days.

### Data sources

Data for exposure, outcomes, and other key covariates were obtained through the EHR. This included data on demographics, comorbidities (using international classification of diseases (ICD)-10 codes), medication use, and blood and urine laboratory test results. Data on cancer type and stage, and occurrence of other IrAE were obtained through ICD-10 codes (online supplemental table S1). All data were obtained from querying Clarity, the relational database underlying the Epic electronic health record (Verona, WI) and we have used this approach in prior publications.^28–31^ We had complete access to person-level data for this study. Death data was obtained from the EHR where it is directly captured for inpatient deaths occurring in our health system and manually entered for outpatient deaths and those occurring outside our health system. On manual chart review of 40 participants, death data accurately matched manual chart review in 98%.

We defined steroid use as at least 20 mg of prednisone or equivalent started within 14 days of AKI. We considered ICI therapy ‘held’ if there were no doses administered within 6 weeks of prior dose and ‘stopped’ if there were no further doses administered. This time window was selected from standard dosing regimens and supported by observed data where >98% of non-held doses were administered within 6 weeks.

### Statistical analysis

We present baseline participant characteristics data as median (IQR) or count (percentage) stratified by mortality, eAIN, and AKI status. We also present characteristics at the time of AKI among those who developed AKI. We report AKI and eAIN incidence rates as events/1000 person-years. Our primary outcome was mortality assessed up to 1 year after initiation of therapy. Both AKI and eAIN were modeled in a time-varying fashion, such that a participant was not defined as being exposed until the time of the event. From that point on, they were considered to have exposure for the duration of follow-up—meaning the estimates associated with AKI or eAIN reflected AKI or eAIN exposure, not necessarily current AKI or eAIN. We only included the first AKI event in our analysis as exposure. Our first set of analyses were Cox proportional hazards models for the outcome of mortality with the primary exposure of interest the first occurrence of AKI or eAIN. The reference group in these analyses was those who did not develop AKI. Follow-up time started at 15 days after the first ICI dose. Our second set of analyses were Cox proportional hazard models for outcome of mortality up to 365 days restricted to patients with AKI. Here, we tested the independent association of eAIN with mortality. In these analyses the reference group was those with AKI who did not have eAIN and follow-up started at the time of AKI. We also tested the association of AKI duration and stage with mortality in those with AKI. For all analyses, we present two models: model 1 tested univariable association of exposure with mortality, whereas model 2 controlled for age, sex, race, ethnicity, and presence of comorbidities (chronic kidney disease (CKD), congestive heart failure, chronic obstructive pulmonary disease, cirrhosis, diabetes, Elixhauser Comorbidity Score), cancer type (lung, melanoma, other), metastasis, baseline creatinine, and type and time-updated administration of ICI. We present data visually using extended Kaplan-Meier curves accounting for time-updated covariate.

We conducted several sensitivity analyses. First, we tested the association of eAIN with mortality where eAIN was defined as those with top 3% of AIN probability based on our diagnostic model. Second, we conducted a landmark analysis where exposure was noted at 90 days after initiation of ICI therapy and held constant thereafter. This analysis only included those who survived at 90 days. Third, we began follow-up at initiation of ICI therapy without excluding the first 15-day period. Fourth, we excluded those participants who received ICI therapy as part of a clinical trial (n=781). We present two models for each of these sensitivity analyses similar to the primary analysis. Finally, we present mortality rates (per 1000-person years) by deciles of predicted probabilities of AIN. We conducted all analyses using STATA (V.15.1) and SAS (V.9.4; SAS Institute).

## Results

### Baseline characteristics

Of the 2373 patients who were initiated on ICI therapy between 2013 and 2019 within the Yale-New Haven Health System, we included 2207 participants after excluding those without available creatinine values before or after ICI initiation (n=70), those without any follow-up information after therapy initiation (n=48) and those who died within 15 days of ICI initiation (n=21, figure 1). Of the 2207 included in the final analysis, 549 (25%) developed AKI and 617 (28%) died within a year of initiation of therapy.

Baseline patient characteristics by mortality status at 1 year are presented in table 1. Notably, as compared with those who survived 1 year after ICI initiation, those who died were similar demographically, though with slightly older age. Patients who died were more likely to have diseases other than melanoma and stage 4 disease. Proton pump inhibitor use was more common at ICI initiation among those who later died than those who survived. Development of AKI was more common in those who died. Many of the irAEs such as dermatitis and thyroiditis were more common in those who survived.

**Table 1:** Characteristics of cohort participants by mortality status at 1 year after immune checkpoint inhibitor therapy initiation

Variables	Died within 365 days (N=617)	Survived >365 days (N=1590)	P value
Baseline			
Demographics			
Age	67.5 (59.4,75.1)	66.1 (57.6,74.8)	0.047
Female sex	271 (43.9%)	688 (43.3%)	0.78
Black race	41 (6.6%)	105 (6.6%)	0.97
Comorbidities			
Hypertension	386 (62.6%)	939 (59.1%)	0.13
Diabetes	139 (22.5%)	316 (19.9%)	0.17
CKD	69 (11.2%)	165 (10.4%)	0.58
Cirrhosis	19 (3.1%)	23 (1.4%)	0.01
Cancer type and stage			
Lung	292 (47.3%)	594 (37.4%)	<0.001
Melanoma	65 (10.5%)	317 (19.9%)	<0.001
Kidney	64 (10.4%)	225 (14.2%)	0.02
Digestive	53 (8.6%)	65 (4.1%)	<0.001
Head and neck	33 (5.3%)	77 (4.8%)	0.62
Breast	10 (1.6%)	57 (3.6%)	0.02
Other	100 (16.2%)	255 (16%)	0.92
Stage 4 cancer	555 (90%)	1267 (79.7%)	<0.001
Laboratory findings			
Creatinine	0.9 (0.7,1.1)	0.9 (0.7,1.1)	0.07
eGFR	83.4 (60.9, 98.2)	82.9 (63.5, 95.6)	0.55
Blood urea nitrogen	16 (12, 20)	16 (12, 21)	0.13
Hemoglobin	11.5 (10.2, 12.8)	12.7 (11.2, 14)	<0.001
Platelet count	264 (194, 345)	243 (195, 306)	<0.001
Bicarbonate	26 (24, 27)	26 (24, 27)	0.65
Medication use			
Antibiotic	357 (57.9%)	853 (53.6%)	0.07
PPI	184 (29.8%)	338 (21.3%)	<0.001
NSAID	152 (24.6%)	332 (20.9%)	0.06
Immune checkpoint inhibitor (ICI)			
Ipilimumab	92 (14.9%)	307 (19.3%)	0.02
Nivolumab	289 (46.8%)	708 (44.5%)	0.33
Pembrolizumab	196 (31.8%)	550 (34.6%)	0.21
Other ICI	100 (16.2%)	257 (16.2%)	0.98
Multiple	75 (12.2%)	265 (16.7%)	0.01
During follow-up			
Acute kidney injury	193 (31.3%)	356 (22.4%)	<0.001
AKI stage 1	92 (14.9%)	225 (14.2%)	0.65
AKI stage 2 or higher	101 (16.4%)	131 (8.2%)	<0.001
Peak creatinine	1.1 (0.8,1.6)	1.1 (0.9,1.5)	0.49
Dialysis	7 (1.1%)	8 (0.5%)	0.11
Immune-related adverse events			
Pneumonitis	55 (8.9%)	54 (3.4%)	<0.001
Adrenalitis	53 (8.6%)	214 (13.5%)	0.002
Colitis	52 (8.4%)	184 (11.6%)	0.03
Dermatitis	46 (7.5%)	243 (15.3%)	<0.001
Hepatitis	17 (2.8%)	58 (3.6%)	0.30
Thyroiditis	16 (2.6%)	91 (5.7%)	0.002
Hypophysitis	14 (2.3%)	91 (5.7%)	0.001
Interstitial nephritis	8 (1.3%)	31 (2.0%)	0.29

Immune-related adverse events defined using ICD codes. Complete list of ICD codes is presented in online supplemental file 1.

AKI, acute kidney injury; CKD, chronic kidney disease; eGFR, estimated glomerular filtration rate; ICD, international classification of diseases; NSAID, non-steroidal anti-inflammatory drug; PPI, proton pump inhibitors.

### AKI and mortality

**Figure 2:**
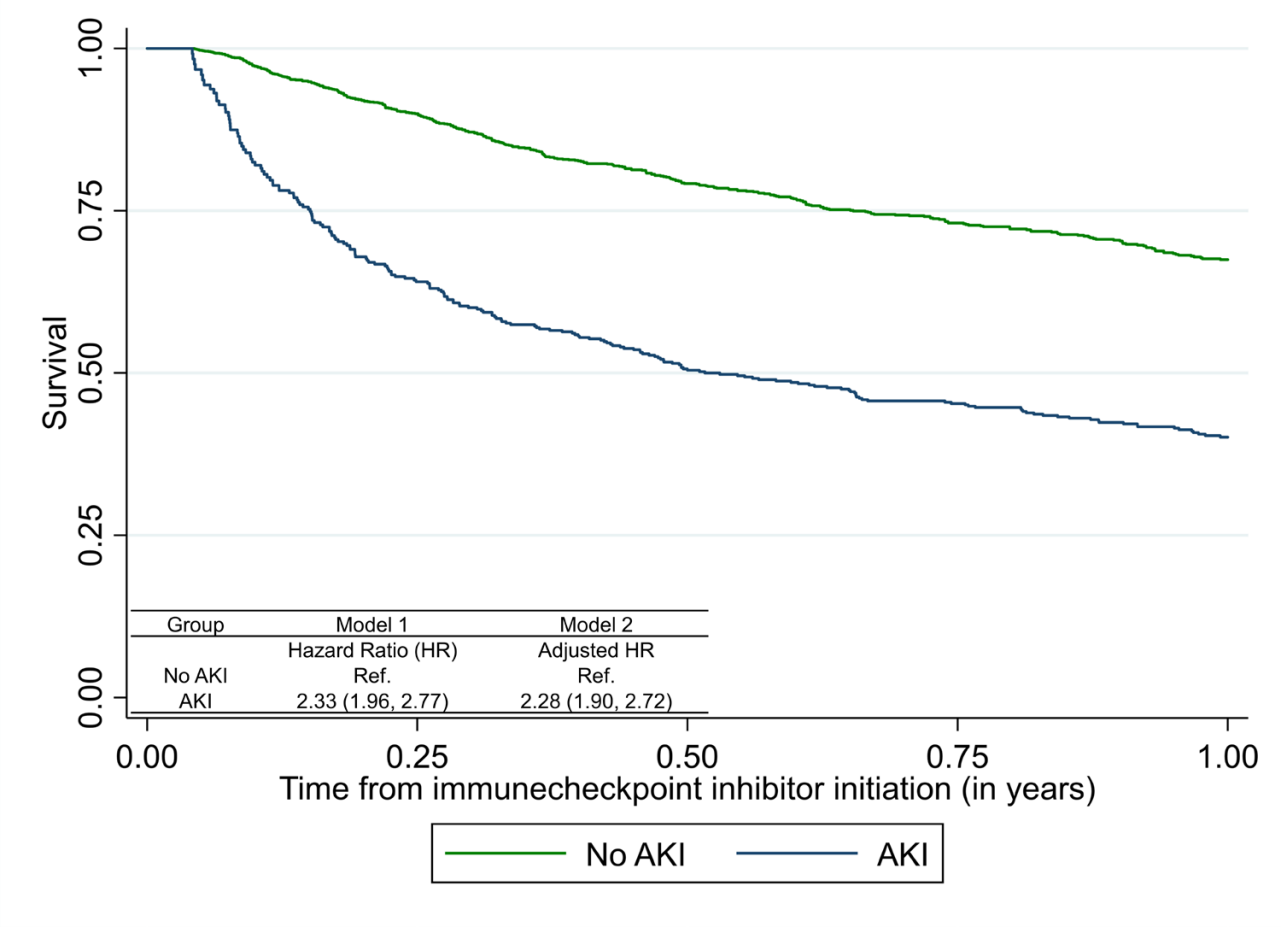
Association of acute kidney injury (AKI) with mortality after immune checkpoint inhibitor therapy. Association of AKI with mortality in all participants initiated on immune checkpoint inhibitor (ICI) therapy using time-varying Cox proportional hazards models where exposure (AKI) was treated as a time-varying covariate updated once if it occurred and patient considered as exposed for the remainder of the analysis period. Follow-up starts 15 days after initiation of immune checkpoint inhibitor therapy for both analyses. Model 1 tests univariable association of AKI with mortality; model 2 controls for age, sex, race, ethnicity, presence of comorbidities (CKD, CHF, COPD, cirrhosis, diabetes, Elixhauser Comorbidity Score), cancer type (lung, melanoma, other), metastasis, baseline creatinine, and time-updated administration of ICI. Extended Kaplan-Meier curve accounting for time-varying covariate. Mortality rate (per 1000 person-years): no AKI, 445 (404, 489); AKI, 905 (786, 1042); overall, 529 (489, 572). CHF, congestive heart failure; CKD, chronic kidney disease; COPD, chronic obstructive pulmonary disease.

A total of 549 (25%) patients developed AKI within the first year of ICI initiation at an incidence rate of 552 per 1000 person-years (online supplemental figure S2). AKI occurred at a median (IQR) of 79.7 (33.5–158.9) days after initiation of ICI therapy (online supplemental table S2). Those who developed AKI tended to be older and more likely to have hypertension and CKD (online supplemental table S3). In time-updated Cox-proportional hazard models, we noted that AKI was associated with higher mortality in fully adjusted analysis (adjusted HR 2.28 (95% CI 1.90 to 2.72), figure 2), and when using an alternate definition of AKI (online supplemental figure S3). There was evidence of non-proportional hazards in the model, such that the association of AKI with mortality was strongest early after AKI and diminished to the background rate at 4 months after AKI (online supplemental figure S4). In patients with AKI, longer duration of AKI was associated with higher mortality (HR 1.84 (95% CI 1.30 to 2.62)) and severe AKI (stage 2 or higher) showed a trend towards higher mortality (HR 1.32 (95% CI 0.98 to 1.79), (online supplemental table S4)).

**Figure 3:**
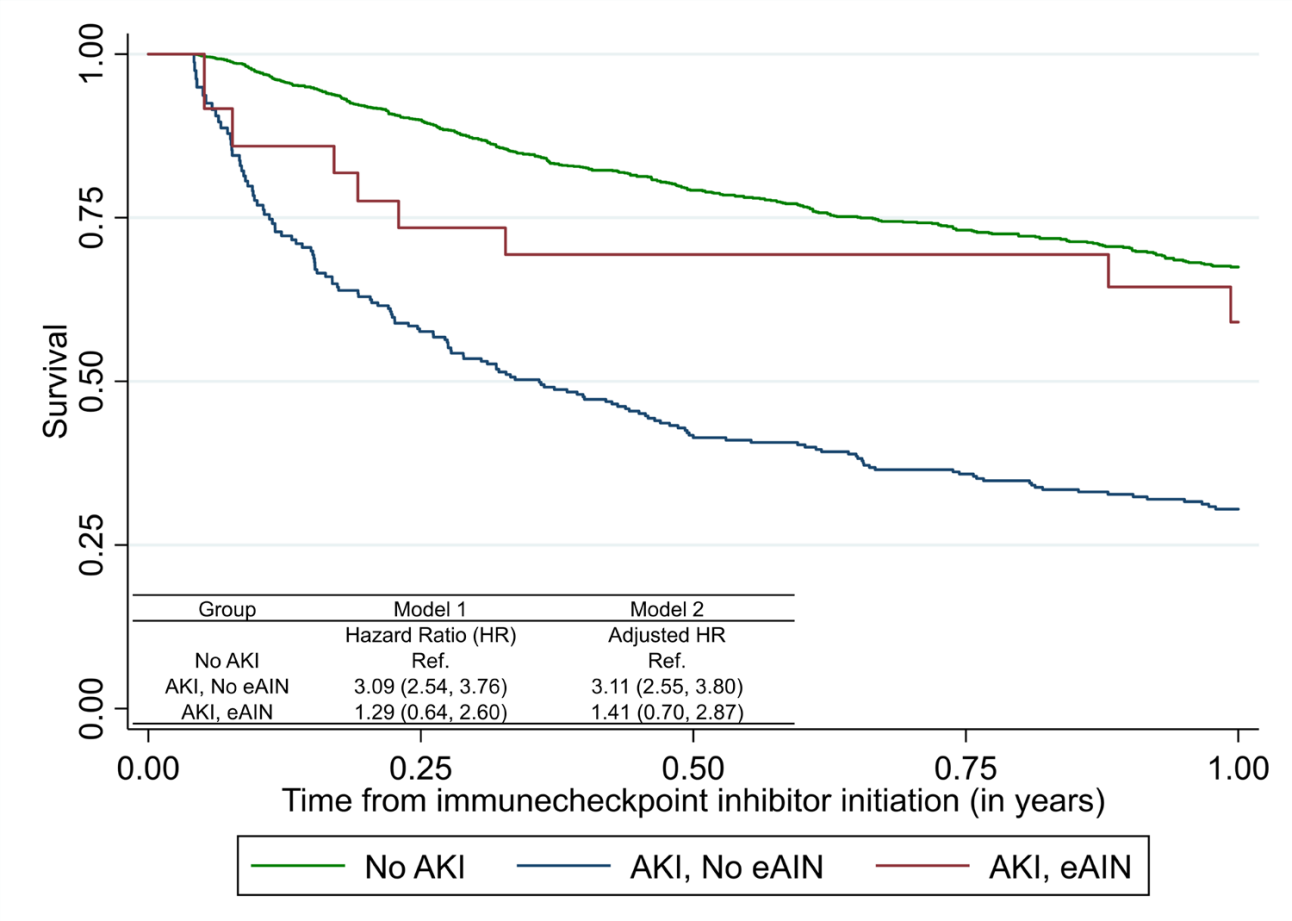
Association of acute interstitial nephritis (AIN) with mortality after immune checkpoint inhibitor therapy. estimated AIN (eAIN) defined as those in the top 10% of AIN probability as determined by the diagnostic model. Model 1 tests univariable association of AKI or eAIN with mortality; model 2 controls for age, sex, race, ethnicity, presence of comorbidities (CKD, CHF, COPD, cirrhosis, diabetes, Elixhauser Comorbidity Score), cancer type (lung, melanoma, other), metastasis, and time-updated administration of immune checkpoint inhibitor (ICI). Association of estimated AIN with mortality in all participants initiated on ICI therapy using time-varying Cox proportional hazards models where exposure (presence or absence of AKI or eAIN) was treated as a time-varying covariate updated once if it occurred and patient considered as exposed for the remainder of the analysis period. Extended Kaplan-Meier curve accounting for time-varying covariate. Follow-up starts at 15 days after initiation of ICI therapy. Mortality rates (per 1000 person-years): no AKI, 498 (452,547); AKI without eAIN, 1487 (1250,1770); AKI with eAIN, 617 (308,1233). CHF, congestive heart failure; CKD, chronic kidney disease; COPD, chronic obstructive pulmonary disease.

**Figure 4:**
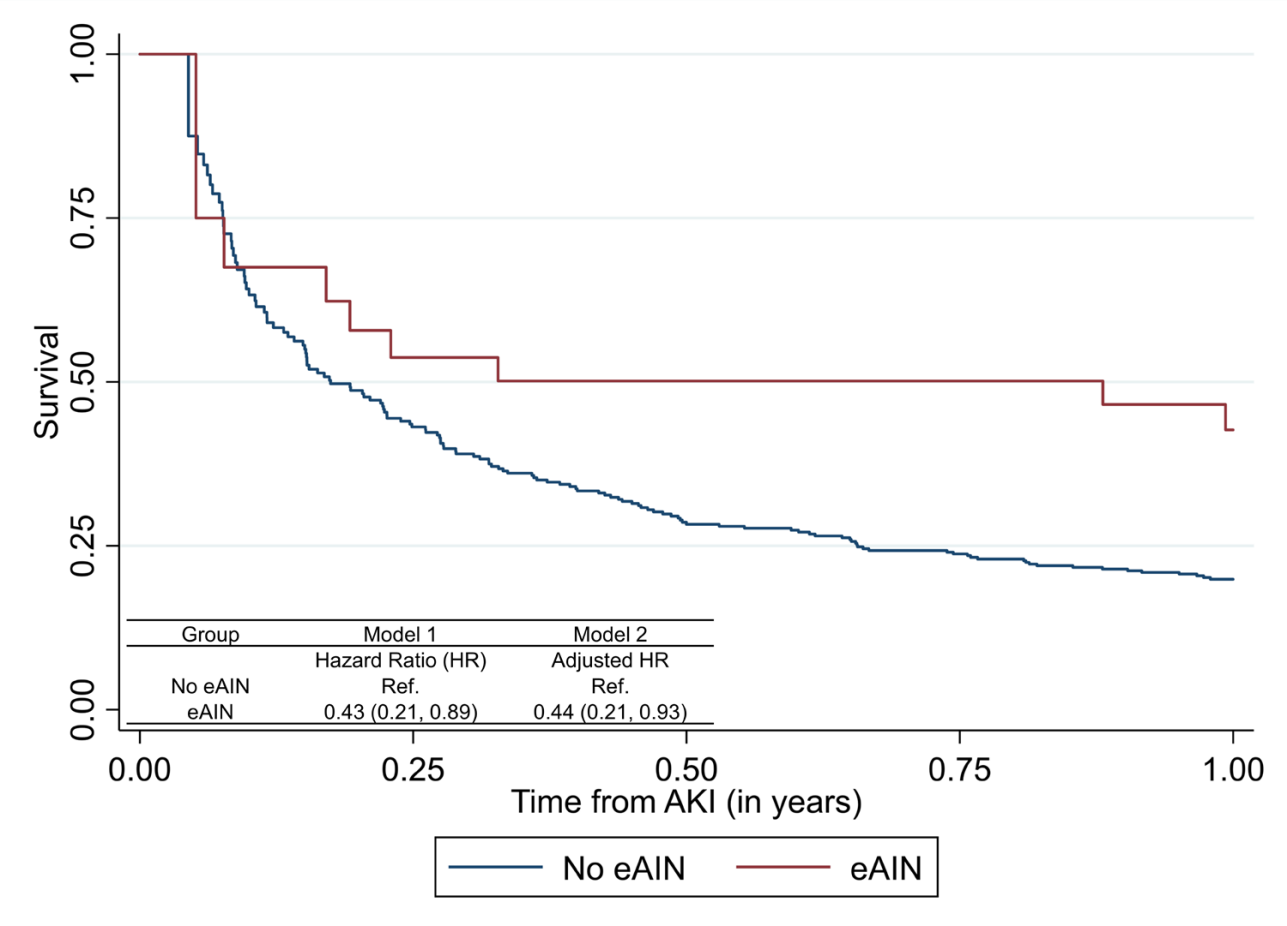
Association of acute interstitial nephritis (AIN) with mortality after immune checkpoint inhibitor (ICI) therapy in those with acute kidney injury. estimated AIN (eAIN) defined as those in the top 10% of AIN probability as determined by the diagnostic model. Model 1 tests univariable association of eAIN with mortality; model 2 controls for age, sex, race, ethnicity, presence of comorbidities (CKD, CHF, COPD, cirrhosis, diabetes, Elixhauser Comorbidity Score), cancer type (lung, melanoma, other), metastasis, and time-updated administration of ICI. Follow-up starts at AKI diagnosis. Association of estimated AIN with mortality among those with AKI using time-varying Cox proportional hazards models where exposure (presence or absence of eAIN) was treated as a time-varying covariate updated once if it occurred and patient considered as exposed for the remainder of the analysis period. Extended Kaplan-Meier curve accounting for time-varying covariate. CHF, congestive heart failure; CKD, chronic kidney disease; COPD, chronic obstructive pulmonary disease.

### Model-eAIN and its clinical correlates

We included 355 (65%) of the 549 patients with AKI in the analysis of estimated AIN (eAIN) and mortality after excluding participants without urinalysis within 7 days of AKI (n=193; online supplemental figure S5). In our primary analysis, we chose to classify thirty-four (10%) of 355 patients in the highest decile of predicted probability of AIN as eAIN based on prior studies that showed 10% prevalence of AIN in those with AKI who underwent a biopsy (table 2). Those with eAIN tended to have more severe AKI as noted by higher serum creatinine (median (IQR), 3.3 (2.1–6.1) vs 1.4 (1.2–1.9) mg/dL) and blood urea nitrogen (39 (25–60) vs 25 (18–37)). Those with eAIN had higher steroid use after AKI (13 (40.6%) vs 62 (22.5%), p=0.02), longer duration to next ICI dose (20.3 (10.2–123.9) vs 10.5 (0.1–20.4) days, p=0.01), and higher proportion with ICD10 codes for interstitial nephritis (17% vs 5%, p=0.006; table 2). We noted a high degree of agreement between model estimated eAIN diagnosis and that determined by nephrologist adjudication, treating clinician impression, and by a recently proposed classification schema (online supplemental table S5). Of the 11 patients who underwent a kidney biopsy to evaluate the underlying cause of AKI, those with AIN on histology (n=8) had a higher predicted probability of AIN based on our model than those with other diagnoses (n=3) (online supplemental table S6).

**Table 2:** Participant characteristics at the time of acute kidney injury by estimated acute interstitial nephritis status

Variables	Estimated acute interstitial nephritis (eAIN; n=34)	Not eAIN (n=321)	P value
Demographics and comorbidities			
Age	66.4 (57.8,75.5)	64.6 (56.7,73.1)	0.52
Female sex	12 (35.3%)	158 (49.1%)	0.13
Black race	1 (2.9%)	20 (6.2%)	0.44
Diabetes	10 (29.4%)	80 (24.8%)	0.56
Hypertension	28 (82.4%)	217 (67.4%)	0.07
Cirrhosis	0 (0%)	7 (2.2%)	0.39
Chronic kidney disease	7 (20.6%)	42 (13%)	0.22
Cancer and stage			
Lung	9 (26.5%)	102 (31.7%)	0.53
Melanoma	5 (14.7%)	46 (14.3%)	0.95
Kidney	10 (29.4%)	57 (17.7%)	0.10
Digestive	1 (2.9%)	34 (10.6%)	0.16
Head and neck	4 (11.8%)	18 (5.6%)	0.16
Breast	0 (0%)	7 (2.2%)	0.39
Other	5 (14.7%)	58 (18%)	0.63
Stage 4 cancer	30 (88.2%)	275 (85.4%)	0.65
Laboratory findings at AKI			
Creatinine	3.3 (2.1, 6.1)	1.4 (1.2, 1.9)	<0.001
Estimated glomerular filtration rate	19.2 (9.3, 26.5)	45.2 (31.8, 59.5)	<0.001
Blood urea nitrogen (BUN)	39 (25, 60)	25 (18, 37)	0.004
BUN:creatinine ratio	11.2 (8.7, 13.9)	16.8 (13.3, 23)	<0.001
Hemoglobin	11.2 (9.6, 12.7)	10.9 (9.2, 12.7)	0.42
Platelet count	237 (173, 360)	238 (172, 317)	0.75
Bicarbonate	22.5 (19, 25)	23 (20, 25)	0.52
Anion gap	16 (14, 19)	15 (12, 17)	0.06
Urine proteinuria	2 (5.9%)	57 (17.7%)	0.13
Urine-specific gravity	1.012 (1.009,1.02)	1.019 (1.013,1.024)	0.002
Diagnostic evaluation of AKI			
Urinalysis	34 (100%)	322 (100%)	N/A
*Clostridium difficile* testing	6 (17.6%)	67 (20.8%)	0.66
Ultrasound	16 (47.1%)	54 (16.8%)	<0.001
Medication use before AKI			
PPI	11 (32.4%)	123 (38.2%)	0.50
NSAID	4 (11.8%)	113 (35.1%)	0.006
Antibiotic	21 (61.8%)	237 (73.6%)	0.14
Medication use after AKI (30 days)			
Steroid use	15 (44.1%)	87 (27%)	0.04
Steroid use (excluding prior use)	13 (40.6%)	62 (22.5%)	0.02
Received ICI after AKI	16 (47.1%)	120 (37.3%)	0.26
Time to next ICI dose, days	20.3 (10.2, 123.9)	10.5 (0.1, 20.4)	0.01
Predicted probability of AIN	0.49 (0.44, 0.56)	0.15 (0.10, 0.25)	<0.001
Immune-related adverse events			
Dermatitis	9 (26.5%)	51 (15.8%)	0.14
Colitis	8 (23.5%)	48 (14.9%)	0.21
Hypophysitis	4 (11.8%)	24 (7.5%)	0.33
Pneumonitis	3 (8.8%)	32 (9.9%)	0.99
Adrenalitis	2 (5.9%)	60 (18.6%)	0.09
Thyroiditis	2 (5.9%)	25 (7.8%)	0.99
Hepatitis	2 (5.9%)	20 (6.2%)	0.99
Interstitial nephritis	6 (17%)	16 (5%)	0.006

Only includes those with AKI and with complete data on components (n=356). Values obtained at AKI.

AIN, acute interstitial nephritis; AKI, acute kidney injury; eAIN, estimated acute interstitial nephritis; ICI, immune checkpoint inhibitor; NSAID, non-steroidal anti-inflammatory drug; PPI, proton pump inhibitor.

### Association of eAIN with mortality

As compared with those without AKI, those with AKI had a threefold higher hazard of death (3.11 (2.55–3.80)), whereas those with eAIN had similar mortality as those without AKI (1.41 (0.70–2.87), figure 3). Among patients with AKI, those with eAIN had lower mortality in both univariable (HR, 0.43 (95% CI 0.21 to 0.89)) and fully adjusted analyses (aHR 0.44 (95% CI 0.21 to 0.93)) than those without eAIN (figure 4). eAIN was independently associated with lower mortality after controlling for AKI stage and duration (online supplemental table S7). The association of individual components of this model with mortality are presented in online supplemental table S8.

### Sensitivity analyses

We conducted several sensitivity analyses to test the robustness of our findings. First, we tested an additional model probability cut-off to define eAIN (3%) and noted similar results as our primary analysis where those with AKI had higher mortality than those without AKI, whereas those with eAIN had similar mortality than those without AKI (online supplemental figure S6). Second, we conducted a landmark analysis where exposure was noted at 90 days after initiation of ICI therapy and held constant thereafter. In this analysis, we only included those who had survived at 90 days and noted similar results as in our primary analysis (online supplemental figure S7). Third, we began follow-up at initiation of ICI therapy without excluding the first 15-day period and noted similar associations of eAIN with mortality (online supplemental figure S8). Fourth, we noted consistent results after excluding those who received ICI therapy as part of a clinical trial (online supplemental figure S9). Finally, we present mortality rates (per 1000-person years) by deciles of predicted probabilities of AIN and noted a lower mortality rate with increasing predicted probability of AIN. For example, those in the lowest decile of predicted probability of AIN had a 5.9-fold higher mortality rate than those with highest decile of probability of AIN (mortality rate, decile 1 vs 10, 4061 (2620, 6295) vs 690 (345, 1381) per 1000 person-years; online supplemental table S9).

## Discussion

In a cohort of patients initiated on ICI therapy, we show that AKI was common, affecting 25% of our patients, and independently associated with higher 1-year mortality. However, among patients with AKI, higher mortality was only noted in those without eAIN. Those with AKI from eAIN had similar mortality as those without AKI. This raises the possibility that, similar to other milder IrAEs, occurrence of kidney IrAE, eAIN, could indicate effectiveness of ICI and therefore improved 1-year survival despite AKI.

AKI has been demonstrated to be an independent risk factor of mortality in many various settings. This is due in small part to the downstream negative effects of AKI, and in large part because declining kidney function commonly occurs alongside the progression of other disease processes. Published studies have demonstrated heterogenous conclusions when examining the relationship of AKI with survival among patients on ICI therapy. For example, a study by García-Carro *et al* found that AKI was an independent risk factor for mortality, whereas that by Meraz-Muñoz *et al* found no association of AKI with mortality.^7 8^ Cortazar *et al* noted higher mortality in those who did not recover kidney function after AKI.^32^ In the current study, using one of the largest cohorts to date, we show that development of AKI after ICI therapy was independently associated with increased mortality. We controlled our analysis for multiple confounders including demographics, comorbidities, cancer characteristics and treatment, and laboratory features. Patients with AKI demonstrated poorer survival rates in the immediate period after the AKI event, with the greatest increase in mortality in the month immediately after AKI, but the increased risk persisted for up to 3 months after AKI. We also noted higher mortality with higher stage and duration of AKI. The biopsy rate in our cohort was extremely low at 1.1%, therefore very few patients had an established diagnosis of AIN to guide treatment. In a recent study, Gupta *et al* showed that corticosteroid use was associated with greater kidney function recovery in a cohort of patients with ICI-associated AKI where 83% of participants had biopsy-proven AIN.^33^

While the most common causes of AKI in the general population include pre-renal azotemia or ATI, ICI-associated AKI also includes AIN, which is considered an IrAE.^34^ We hypothesized that the association of AKI with mortality would vary based on its underlying etiology such that those with kidney IrAE would have lower mortality compared with those with other causes of AKI. Low-grade IrAEs affecting the dermatological and endocrine systems have been associated with improved survival in patients on ICIs^1 35–43^ since they may indicate host immune activation, the same mechanism by which ICIs exert their effective antitumor responses. While it is challenging to differentiate between AKI etiologies in clinical and research settings and only a kidney biopsy or biomarkers can definitively differentiate between these etiologies, this procedure carries bleeding risk^44–48^ and is usually foregone in patients on ICIs in favor of empiric corticosteroid treatment. Therefore, to test our hypothesis, we used an externally validated AIN diagnostic model to assign participants’ likelihood of AIN.^23^ This score was developed at our institution and externally validated at Indiana University in over 1500 participants with histological diagnosis and showed an AUC of 0.74 (0.69, 0.79) for biopsy-proven AIN diagnosis in external validation. In the current study, those with a high probability of AIN tended to have greater steroids use after AKI as well as longer duration between AKI and subsequent ICI dose, both of which indicate a higher suspicion of IrAE by the treating clinician. Moreover, among those who underwent a kidney biopsy, those with AIN had higher predicted probability of AIN based on the diagnostic model than those without AIN. We also found high concordance between model-predicted eAIN and that determined by an adjudicating nephrologist, opinion documented by the treating clinician, and that determined through a recently proposed algorithm.^10^

We noted that those in the top decile of AIN probability based on our model, referred to as eAIN, had more severe AKI as noted by greater rise in serum creatinine and blood urea nitrogen than those without eAIN. Despite this, those with eAIN had a lower mortality compared with those without eAIN. Moreover, while patients without eAIN had a threefold higher mortality than those without AKI, those with eAIN had similar mortality as those without AKI. These findings remained consistent across various sensitivity analyses including defining eAIN as top 3% of predicted probability of AIN and performing a landmark analysis at 3 months. Thus, patients with eAIN reflect a unique phenotype not entirely reflected by rise in creatinine. The theoretical basis for AIN to serve as a biomarker of ICI efficacy and our finding that patients with eAIN had improved mortality compared with all patients with AKI provide preliminary data to support better differentiation between various etiologies of AKI,^49^ both clinically and when using large datasets to answer important questions.

Our study has several clinical and research implications. Clinically, patients who develop ICI-associated AKI represent a cohort of patients with a higher risk of mortality as compared with patients who receive ICI therapy and do not develop AKI. However, our data suggest that the prognosis after AKI may be modified by the underlying etiology of AKI. Therefore, patients who develop ICI-associated AKI should undergo testing, when clinically appropriate, to determine whether the underlying cause of AKI is AIN. Not only is the management of this cause of AKI different from others, but it also provides additional prognostic information. Future research studies could prospectively enroll participants on ICI therapy and evaluate AIN biomarkers such as interleukin-9 and tumor necrosis factor-α to differentiate ATI from AIN with greater certainty.^26 30^ Future studies could also evaluate the impact of rapid reintroduction of ICI therapy in those with AKI due to AIN given that these therapies have significant mortality benefit and our preliminary data shows no significant increase in mortality in those predicted to have AIN.

This study has several strengths. First, to the best of our knowledge, this is the largest dataset published to date examining various etiologies of AKI after ICI use using a validated, EHR-based score, which has significant advantages over past studies. While other studies have used biopsy-proven cases of AKI, such an approach introduces significant selection bias as only those with the severest forms of AKI undergo a biopsy and most cases of AKI are managed without a biopsy. Our study included time-to-event analyses with 12 months of follow-up and we controlled for several confounders including age, comorbidities, cancer and ICI type. Importantly, however, our analyses did not consider a number of other cancer-associated prognostic factors including use of concurrent anti-cancer treatments, treatment line of ICI, patient performance status and molecular/genomic biomarkers. Despite the expected heterogeneity in our cohort due to these factors, we consider that our cohort comprising a large number of consequent patients treated in a multi-institutional healthcare system would reflect the real-world outcomes of patients treated with ICIs. Limitations of our work include its retrospective nature and that the analysis was performed using data derived from a single regional healthcare system. However, our healthcare system includes seven hospitals and associated outpatient practices which includes community and university centers, teaching and non-teaching hospitals, as well as rural and urban settings. Although we have not followed up patients beyond the 12 month cut-off, we believe that our strategy limiting follow-up duration to 12 months would further strengthen the associations suggested between AKI and clinical outcomes. We also lacked biopsy or biomarker data to definitively distinguish between different types of AKI in this study, which could have led to some misclassification of AKI etiology. We did not use chart review in all patients to determine the exact cause of AKI, because in the absence of biopsy data clinical adjudication cannot reliably differentiate between ATI and AIN. However, chart review in a subset of participants showed high concordance between model-predicted AIN and that by adjudicating nephrologist, treating clinician, and a proposed schema for diagnosing AIN in patients on ICI. We also used ICD codes rather than clinician adjudication to determine IrAEs which may have led to some misclassification of IrAEs. Another limitation of our study was excluding any recurrent AKI events, which could have led to misclassification of exposure if subsequent AKI events had a different AIN probability than the initial event. Due to limitations of sample size, we could not test our findings in specific cancer types. Death data were obtained directly through the EHR which could have led to misclassification of the outcome status. However, we confirmed the accuracy through direct chart review in 98% of participants. Finally, our findings could not address whether prescribing corticosteroids or holding ICI therapy after AKI were associated with better outcomes.

In conclusion, we noted that the occurrence of AKI was independently associated with lower survival of patients treated with ICI, particularly in the first 120 days of its occurrence. However, among patients with AKI, those with AIN had higher survival than those without AIN indicating that this IrAE may be a marker of therapeutic response to ICI. Future studies could consider further phenotyping AKI cases using biomarkers and biopsy data to better understand the relationship of various etiologies of ICI-associated AKI with mortality.

## Data Availability

Data are available on reasonable request.
