# Supplementary material for: Mortality after acute kidney injury and acute interstitial nephritis in patients prescribed immune checkpoint inhibitor therapy
Source: J Immunother Cancer. 2022 Mar 29;10(3):e004421. doi: 10.1136/jitc-2021-004421 (PMC8968986; doi:10.1136/jitc-2021-004421)
Supplement: Supplementary data [file jitc-2021-004421supp001.pdf]

SUPPLEMENTAL FILE

Mortality after acute kidney injury and acute interstitial nephritis in patients prescribed immune checkpoint inhibitor therapy

Baker et. al. 2022 Journal for ImmunoTherapy of Cancer

Contents

Table S1. ICD10 codes used to define immune-related adverse events 2
Table S2. Participant characteristics at the time of AKI 3
Table S3. Baseline characteristics by AKI status 4
Table S4. Association of acute kidney injury duration and stage with mortality after immune checkpoint inhibitor therapy 5
Table S5. Concordance between model predicted diagnosis and that obtained by various other methods. 6
Table S6. AIN probability by histological diagnosis 7
Table S7. Association of estimated acute interstitial nephritis with mortality controlling for AKI stage and duration 8
Table S8. Association of individual components of AIN score with mortality after AKI 9
Table S9. Mortality rate by deciles of predicted probability of AIN 10
Figure S1. Components of AIN probability model 11
Figure S2. Incidence of AKI over time 12
Figure S3. Association of alternate AKI definition with mortality after immune checkpoint inhibitor therapy 13
Figure S4. Risk of mortality after AKI over time 14
Figure S5. Flow diagram of mortality by estimated acute interstitial nephritis 15
Figure S6. Association of eAIN with mortality where eAIN was defined as those with the top 3% AIN probability 16
Figure S7. Association of eAIN with mortality using landmark analysis set at 90 days after initiation of immune checkpoint inhibitors 17
Figure S8. Association of eAIN with mortality without excluding first 15 days after initiation of immune checkpoint inhibitors 18
Figure S9. Association of eAIN with mortality after excluding those who received immune checkpoint inhibitor therapy as part of a clinical trial 19

Baker ML, *et al. J Immunother Cancer* 2022; 10:e004421. doi: 10.1136/jitc-2021-004421

Table S1. ICD10 codes used to define immune-related adverse events

**Irae**	**Icd10_code**
**Pnuemonitis**	J69.0, J69.8
**Hepatitis**	K75.0, K75.3, K75.4, K75.81, K75.89, K75.9
**Adrenalitis**	E27.0, E27.1, E27.2, E27.3, E27.40, E27.49, E27.8, E27.9
**Dermatitis**	L20.84, L20.9, L21.8, L21.9, L23.1, L23.2, L23.5, L23.9, L24.4, L24.5, L24.89, L24.9, L25.1, L25.5, L25.8, L25.9, L27.0, L27.1, L27.2, L30.0, L30.1, L30.4, L30.8, L30.9
**Hypophysitis**	E23.0, E23.1, E23.3, E23.6, E23.7
**Thyroiditis**	E06.0, E06.1, E06.2, E06.3, E06.4, E06.5, E06.9
**Colitis**	K52.0, K52.1, K52.81, K52.82, K52.831, K52.839, K52.89, K52.9
**Nephritis**	N10, N11.8, N11.9, N12

Table S2. Participant characteristics at the time of AKI

**Variables**	**Result**
Total with AKI (in the first year after starting ICI)	549/2207
AKI characteristics	
Time to First AKI, days	79.7 (33.5,158.9)
Duration of AKI, days	6.5 (1.5,15.1)
Duration of AKI > 7 Days	280 (51%)
Stage 1	317 (57.7%)
Stage 2 or Higher	232 (42.3%)
Dialysis	15 (2.7%)
Diagnostic evaluation of AKI	
Urinalysis	356 (64.8%)
Clostridium Difficile Testing	81 (14.8%)
Ultrasound	75 (13.7%)
Medication use before AKI (30 days)	
PPI	62 (11.3%)
NSAID	32 (5.8%)
Cisplatin	14 (2.6%)
Medication use after AKI (30 days)	
Steroid after AKI - Overall	126 (23%)
Steroid after AKI - Stage 1	52 (16%)
Steroid after AKI - Stage 2 or Higher	74 (32%)
Time to Steroid	2.5 (0.5,9.9)
Steroid after AKI - Overall (Excluded Prior use)	93 (16.9%)
Steroid after AKI - Stage 1 (Excluded Prior use)	38 (12%)
Steroid after AKI - Stage 2 or Higher (Excluded Prior use)	55 (24%)
Time to Steroid (Excluded Prior use)	4 (1,12.6)
Received ICI after AKI	255 (46.4%)
Time to Next ICI Dose, weeks	7 (0.1,16.5)
Other immune related adverse events	
Pneumonitis	1 (0.2%)
Autoimmune hepatitis	11 (2%)
Adrenalitis	10 (1.8%)
Dermatitis	22 (4%)
Hypophysitis	41 (7.5%)
Thyroiditis	25 (4.6%)
Colitis	91 (16.6%)

Table S3. Baseline characteristics by AKI status

Variables	AKI (N=549)	No AKI (N=1658)	P value
**Demographics**			
Age	65.4 (57.3,73.3)	67 (58.5,75.1)	0.02
Female sex	250 (45.5%)	709 (42.8%)	0.26
Black race	35 (6.4%)	111 (6.7%)	0.79
**Comorbidities**			
Diabetes	129 (23.5%)	326 (19.7%)	0.05
Hypertension	364 (66.3%)	961 (58%)	<0.001
Cirrhosis	11 (2%)	31 (1.9%)	0.84
CKD	71 (12.9%)	163 (9.8%)	0.04
**Cancer type and stage**			
Lung	196 (35.7%)	690 (41.6%)	0.01
Melanoma	88 (16%)	294 (17.7%)	0.36
Kidney	87 (15.8%)	202 (12.2%)	0.03
Digestive	35 (6.4%)	75 (4.5%)	0.08
Head and neck	8 (1.5%)	59 (3.6%)	0.01
Breast	41 (7.5%)	77 (4.6%)	0.01
Other	94 (17.1%)	261 (15.7%)	0.45
Stage 4 cancer	465 (84.7%)	1357 (81.8%)	0.13
**Laboratory findings**			
Creatinine	0.8 (0.7,1)	0.9 (0.7,1.1)	<0.001
eGFR	86.3 (65.5,99.3)	81.8 (62.2,95.2)	0.0005
BUN	16 (12,21)	16 (12,20)	0.66
BUN:Creatinine ratio	18.8 (14.5,23.5)	17.6 (14,22.2)	0.002
Hemoglobin	12 (10.4,13.5)	12.4 (10.9,13.8)	<0.001
Platelet count	257 (198,334)	245 (194,310)	0.01
Bicarbonate	26 (24,27)	26 (24,27)	0.47
Anion gap	13 (11,14)	12 (11,14)	0.03
**Medication use**			
PPI	140 (25.5%)	382 (23%)	0.24
NSAID	129 (23.5%)	355 (21.4%)	0.31
Antibiotic	314 (57.2%)	896 (54%)	0.20
**Immune checkpoint inhibitor (ICI)**			
Ipilimumab	118 (21.5%)	281 (16.9%)	0.02
Nivolumab	268 (48.8%)	729 (44%)	0.05
Pembrolizumab	164 (29.9%)	582 (35.1%)	0.02
Other ICI	86 (15.7%)	271 (16.3%)	0.71
Combination of ICI use	96 (17.5%)	244 (14.7%)	0.12

Table S4. Association of acute kidney injury duration and stage with mortality after immune checkpoint inhibitor therapy

Predictor	Hazard ratio (HR)	Adjusted HR
AKI duration		
≤7days	Ref	Ref
>7days	2.12 (1.51, 2.98)	1.84 (1.30, 2.62)
AKI stage		
Stage 1	Ref	Ref
Stage 2 or 3	1.28 (0.96, 1.71)	1.32 (0.98, 1.79)

Model 1 tests univariable association of AKI with mortality
Model 2 controls for age, sex, race, ethnicity, presence of comorbidities (CKD, CHF, COPD, cirrhosis, diabetes, Elixhauser comorbidity score), cancer type (lung, melanoma, other), metastasis, baseline creatinine, and time-updated administration of ICI.
Follow-up starts at AKI.

Table S5. Concordance between model predicted diagnosis and that obtained by various other methods

Diagnosis method	Agreement	Kappa
**Nephrologist adjudication**	90%	0.80 (P<0.001)
**Treating clinician impression**	78%	0.59 (P=0.008)
**Gupta/Leaf classification**	85%	0.70 (P<0.001)

Data from 20 participants from the highest and lowest deciles of AIN probability. Chart reviewed by a board-certified nephrologist to determine their probability of AIN and collect information on treating oncologist's presumed etiology of AKI. We also determined etiology of AKI based on a schema proposed by Gupta and Leaf (Kidney 360 2020 DOI: https://doi.org/10.34067/KID.0000852019).

Table S6. AIN probability by histological diagnosis

Characteristic	AIN on histology (n=8)	Other diagnosis (n=3)	P-value
**AIN probability of model**	0.30 (0.26, 0.37)	0.13 (0.10, 0.20)	0.03

**excludes kidney biopsy performed to evaluate renal mass*

Baker ML, *et al. J Immunother Cancer* 2022; 10:e004421. doi: 10.1136/jitc-2021-004421

Table S7. Association of estimated acute interstitial nephritis with mortality controlling for AKI stage and duration

**Predictor**	**Adjusted hazard ratio (95% CI)**	
	**Model1**	**Model2**
eAIN vs. no eAIN	0.40 (0.18, 0.91)	0.39 (0.18, 0.83)

Both analyses control for age, sex, race, ethnicity, presence of comorbidities (CKD, CHF, COPD, cirrhosis, diabetes, Elixhauser comorbidity score), cancer type (lung, melanoma, other), metastasis, and time-updated administration of ICI. Model 1 additionally controls for AKI stage and model 2 additionally controls for AKI duration.

Baker ML, *et al. J Immunother Cancer* 2022; 10:e004421. doi: 10.1136/jitc-2021-004421

Table S8. Association of individual components of AIN score with mortality after AKI

Component	Hazard ratio	Adjusted Hazard ratio
Creatinine*	1.14 (0.82, 1.56)	1.40 (0.97, 2.01)
BUN to creatinine ratio*	**4.60 (2.88, 7.35)**	**4.98 (3.05, 8.12)**
Urine specific gravity	**1.02 (1.01, 1.03)**	1.00 (0.99, 1.02)
Urine protein, >=2+	**1.63 (1.09, 2.42)**	**1.70 (1.12, 2.59)**

**per log increase; multivariable model adjusts for each component of the score; follow-up starts at AKI*

Table S9. Mortality rate by deciles of predicted probability of AIN

AIN probability Decile	AIN probability	Mortality rate (95% CI)
**1**	0.00-0.06	4061 (2620,6295)
**2**	0.06-0.09	2020 (1255,3249)
**3**	0.09-0.11	1893 (1193,3004)
**4**	0.11-0.14	1485 (843,2614)
**5**	0.14-0.17	1549 (934,2570)
**6**	0.17-0.22	1036 (589,1825)
**7**	0.22-0.25	751 (391,1443)
**8**	0.25-0.31	1149 (652,2023)
**9**	0.31-0.39	1279 (726,2252)
**10**	0.40-0.75	690 (345,1381)

Mortality rate after AKI per 1000 person-years

Figure S1. Components of AIN probability model

```
EHR model= (Log serum creatinine at AKI* 0.8367518) + (Log blood urea
nitrogen to creatinine ratio* -0.9181938)) + ((Urine dipstick specific
gravity-1)*1000*(-0.0501203)) + (Urine dipstick protein*0. 9360127) +
0.770398
```

Serum creatinine (in mg/dl), blood urea nitrogen to creatinine ratio (dimensionless), dipstick urine specific gravity (dimensionless) and dipstick urine protein coded as 1+ or lower=1 and 2+ or higher=0. Probability calculated as (probability of AIN)=e^(EHR model)/(1+e^(EHR model))

Figure S2. Incidence of AKI over time

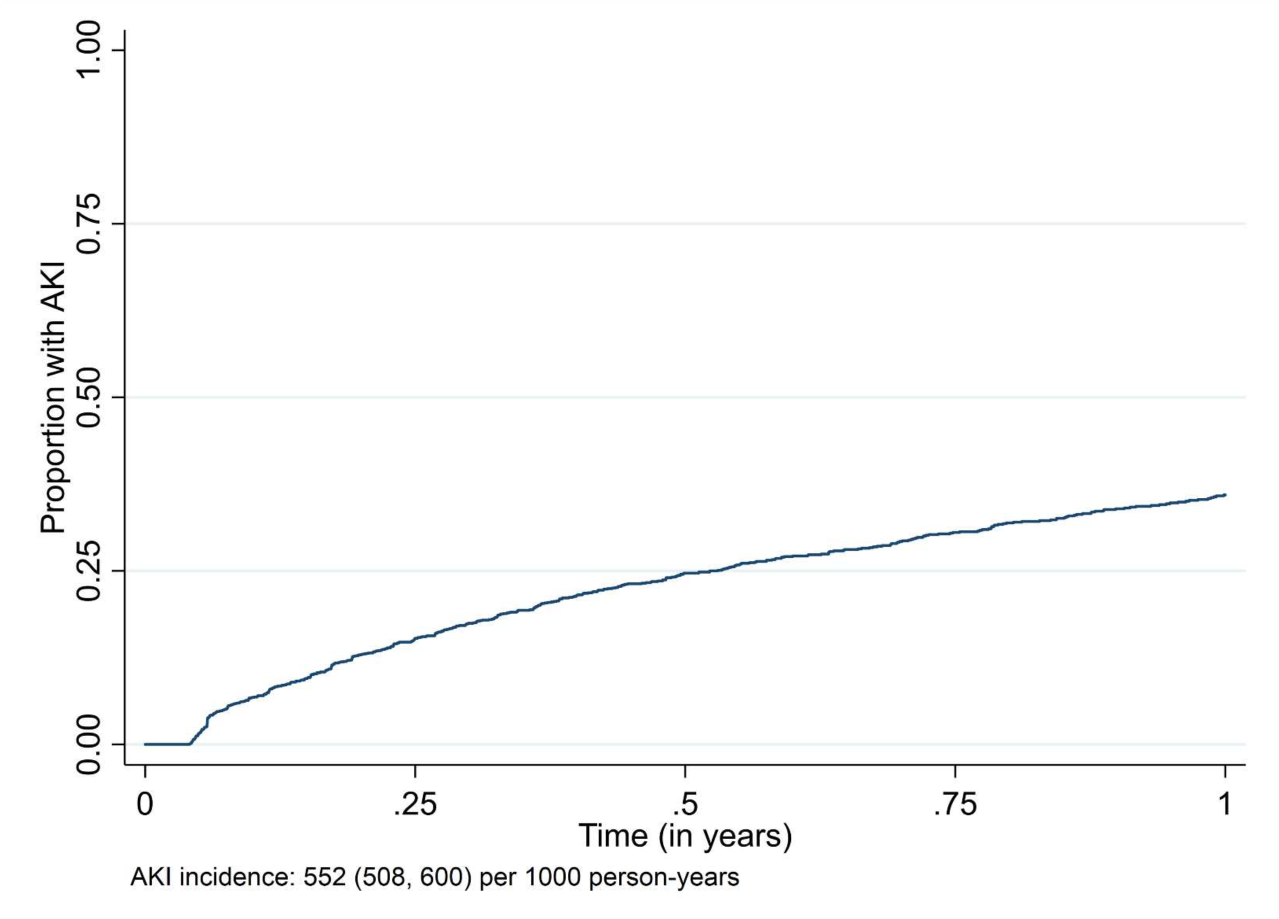

Figure S3. Association of alternate AKI definition with mortality after immune checkpoint inhibitor therapy

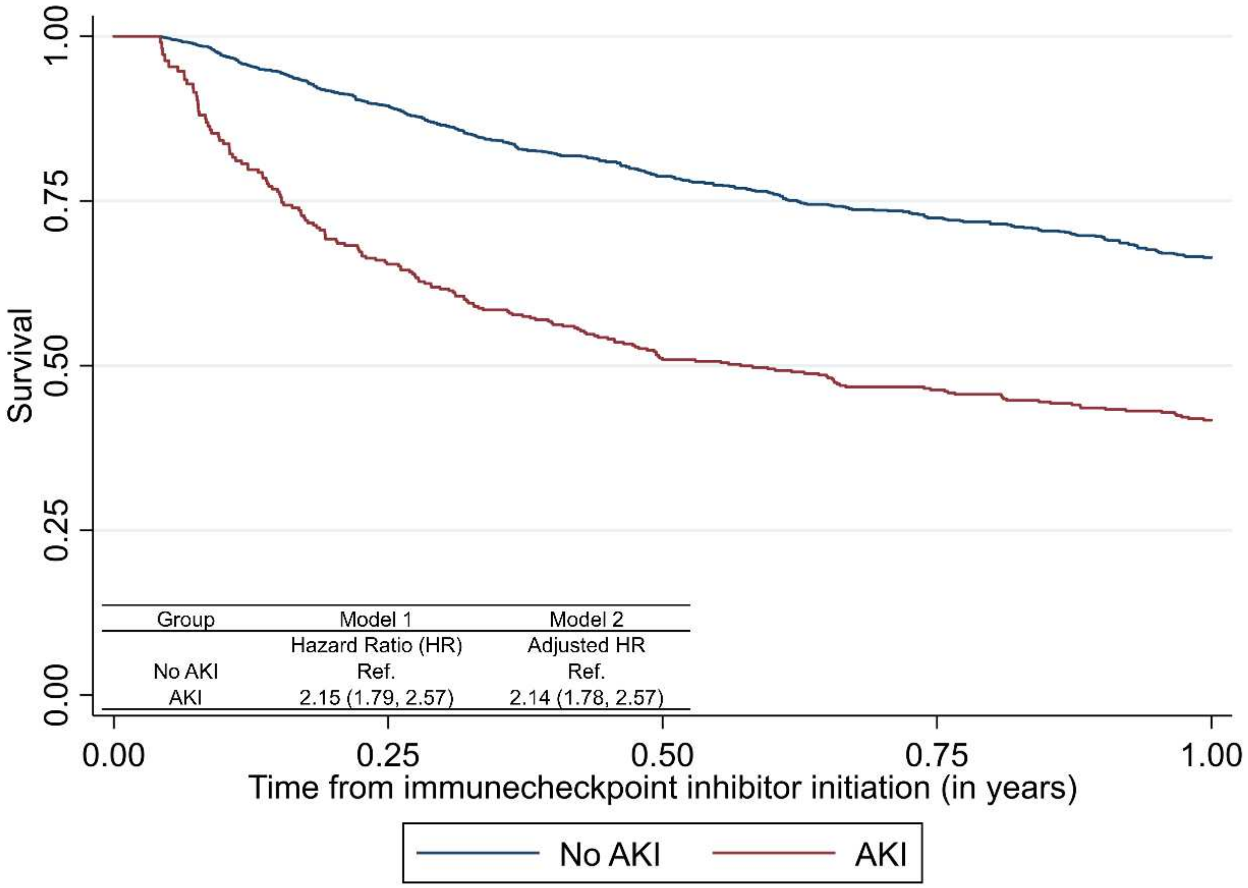

- *Association of AKI with mortality in all participant using time-varying Cox proportional hazards models where exposure (presence or absence of AKI) was treated as a time-varying covariate updated once if it occurred and patient considered as exposed for the remainder of the analysis period*
- *Follow-up starts at 15 days after initiation of immune-checkpoint inhibitor therapy*
- *AKI defined as 50% increase in creatinine over mean of all serum creatinine values obtained within 6 months prior to ICI initiation.*
- *Model 1 tests univariable association of AKI with mortality; Model 2 controls for age, sex, race, ethnicity, presence of comorbidities (CKD, CHF, COPD, cirrhosis, diabetes, Elixhauser comorbidity score), cancer type (lung, melanoma, other), metastasis, and time-updated administration of ICI.*

.

Figure S4. Risk of mortality after AKI over time

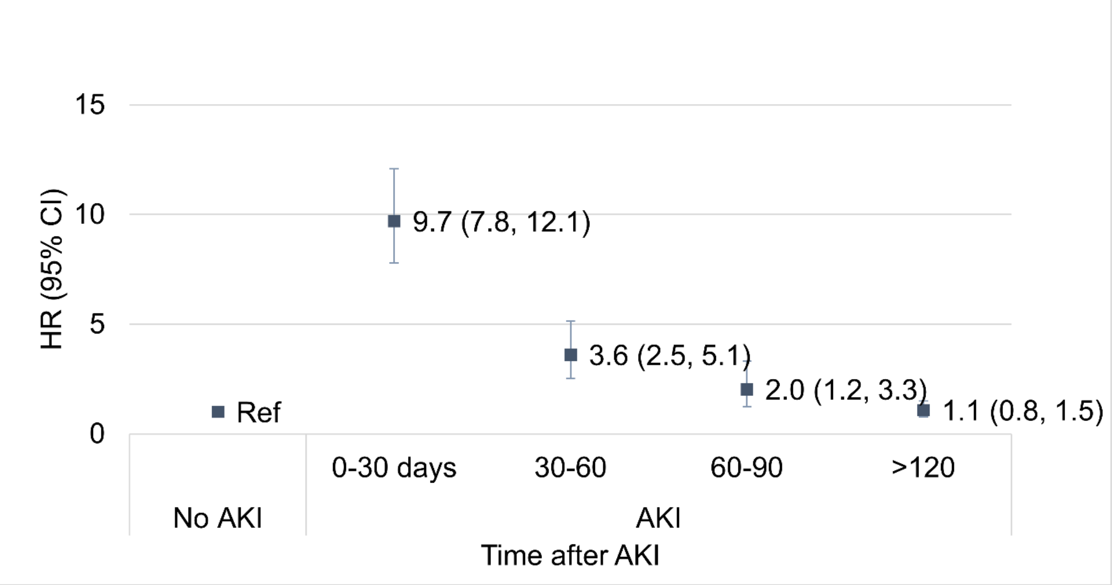

14

Figure S5. Flow diagram of mortality by estimated acute interstitial nephritis

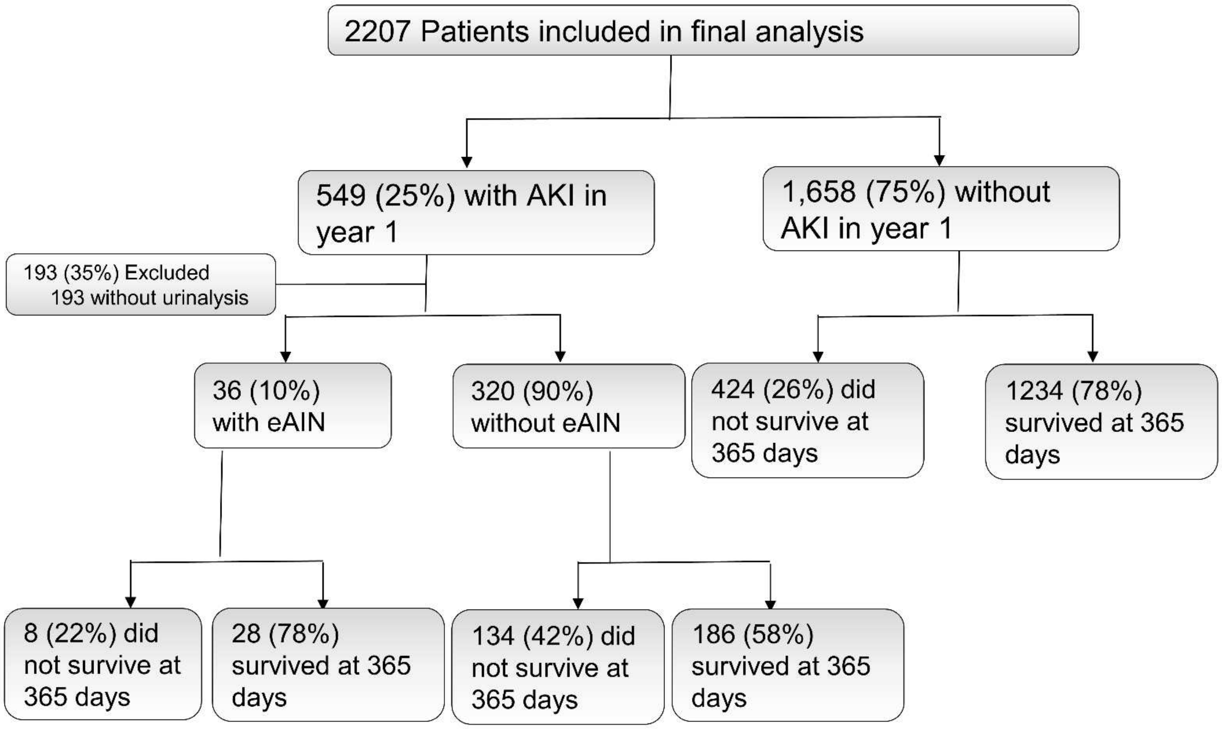

**26% mortality among those with missing UA*

Figure S6. Association of eAIN with mortality where eAIN was defined as those with the top 3% AIN probability

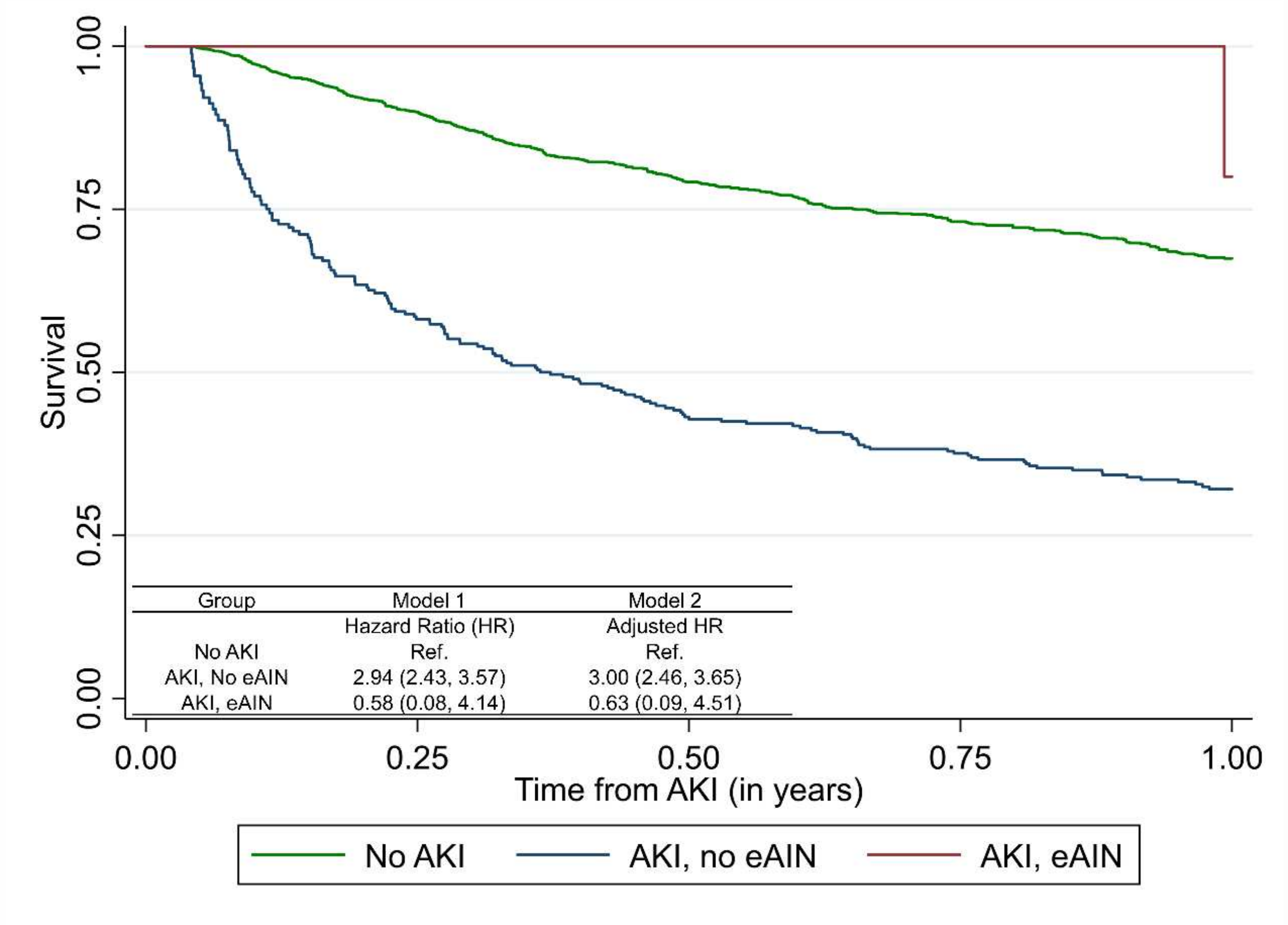

- *Association of estimated AIN with mortality in all participants initiated on immune-checkpoint inhibitor therapy using time-varying Cox proportional hazards models where exposure (presence or absence of AKI or eAIN) was treated as a time-varying covariate updated once if it occurred and patient considered as exposed for the remainder of the analysis period.*
- *Follow-up starts at 15 days after initiation of immune-checkpoint inhibitor therapy*
- *eAIN defined as those in the top 3% of AIN probability as determined by the diagnostic model.*
- *Model 1 tests univariable association of AKI with mortality; Model 2 controls for age, sex, race, ethnicity, presence of comorbidities (CKD, CHF, COPD, cirrhosis, diabetes, Elixhauser comorbidity score), cancer type (lung, melanoma, other), metastasis, and time-updated administration of ICI.*
- *Extended Kaplan Meier curve accounting for time-varying covariate.*

Figure S7. Association of eAIN with mortality using landmark analysis set at 90 days after initiation of immune checkpoint inhibitors

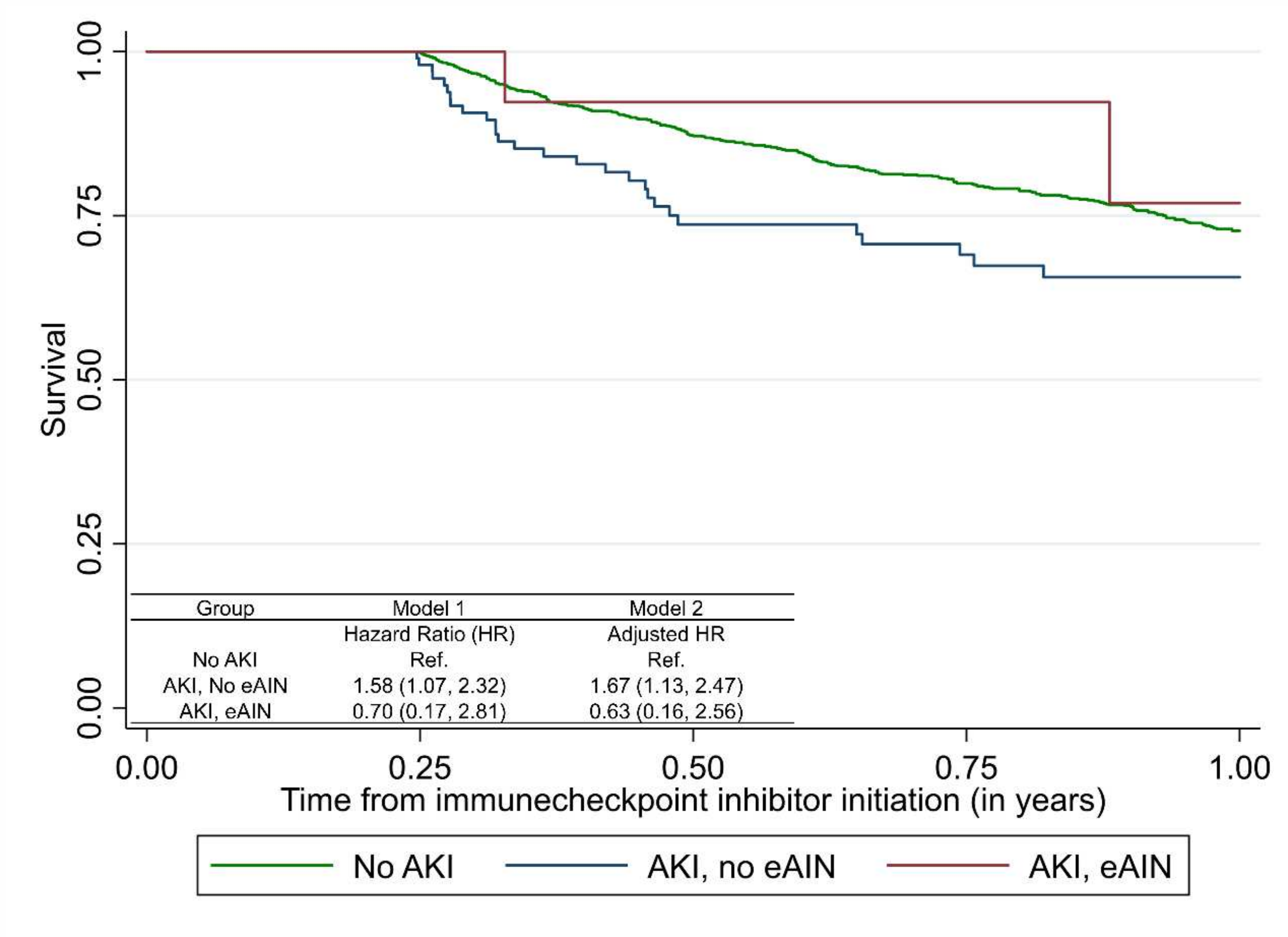

- *Association of estimated AIN with mortality in all participants initiated on immune-checkpoint inhibitor therapy using landmark analysis using Cox proportional hazards models where exposure (presence or absence of AKI or eAIN) was determined at 90 days and held constant for the remainder of the analysis period.*
- *Follow-up starts at 90 days after initiation of immune-checkpoint inhibitor therapy. Only includes those who survived till 90 days after initiation of ICI therapy.*
- *eAIN defined as those in the top 10% of AIN probability as determined by the diagnostic model.*
- *Model 1 tests univariable association of AKI with mortality; Model 2 controls for age, sex, race, ethnicity, presence of comorbidities (CKD, CHF, COPD, cirrhosis, diabetes, Elixhauser comorbidity score), cancer type (lung, melanoma, other), metastasis, and time-updated administration of ICI.*
- *Extended Kaplan Meier curve accounting for time-varying covariate.*

Figure S8. Association of eAIN with mortality without excluding first 15 days after initiation of immune checkpoint inhibitors

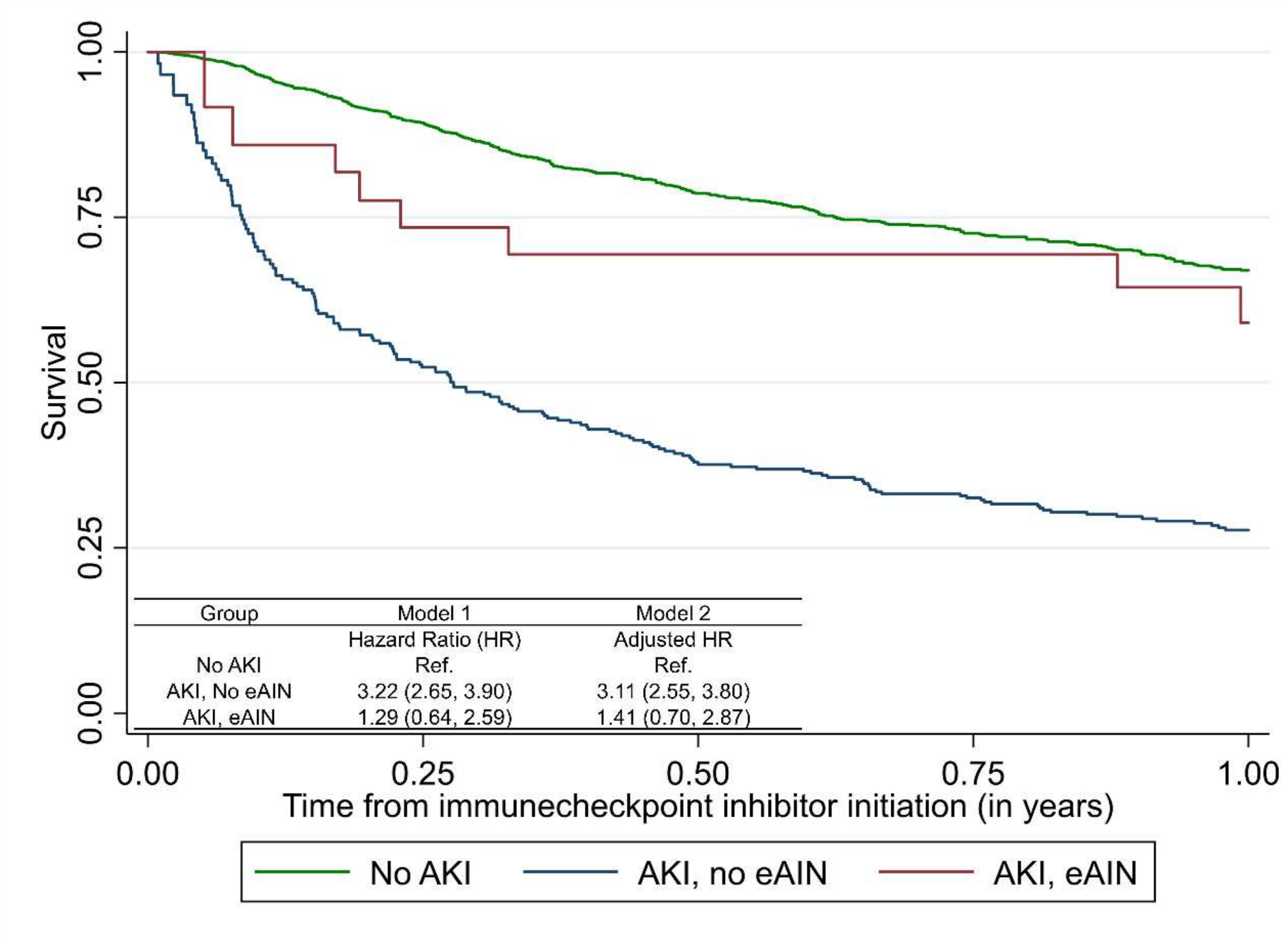

- *Association of estimated AIN with mortality in all participants initiated on immune-checkpoint inhibitor therapy using time-varying Cox proportional hazards models where exposure (presence or absence of AKI or eAIN) was treated as a time-varying covariate updated once if it occurred and patient considered as exposed for the remainder of the analysis period.*
- *Follow-up starts at initiation of immune-checkpoint inhibitor therapy*
- *eAIN defined as those in the top 10% of AIN probability as determined by the diagnostic model.*
- *Model 1 tests univariable association of AKI with mortality; Model 2 controls for age, sex, race, ethnicity, presence of comorbidities (CKD, CHF, COPD, cirrhosis, diabetes, Elixhauser comorbidity score), cancer type (lung, melanoma, other), metastasis, and time-updated administration of ICI.*
- *Extended Kaplan Meier curve accounting for time-varying covariate.*

Baker ML, *et al. J Immunother Cancer* 2022; 10:e004421. doi: 10.1136/jitc-2021-004421

Figure S9. Association of eAIN with mortality after excluding those who received immune checkpoint inhibitor therapy as part of a clinical trial

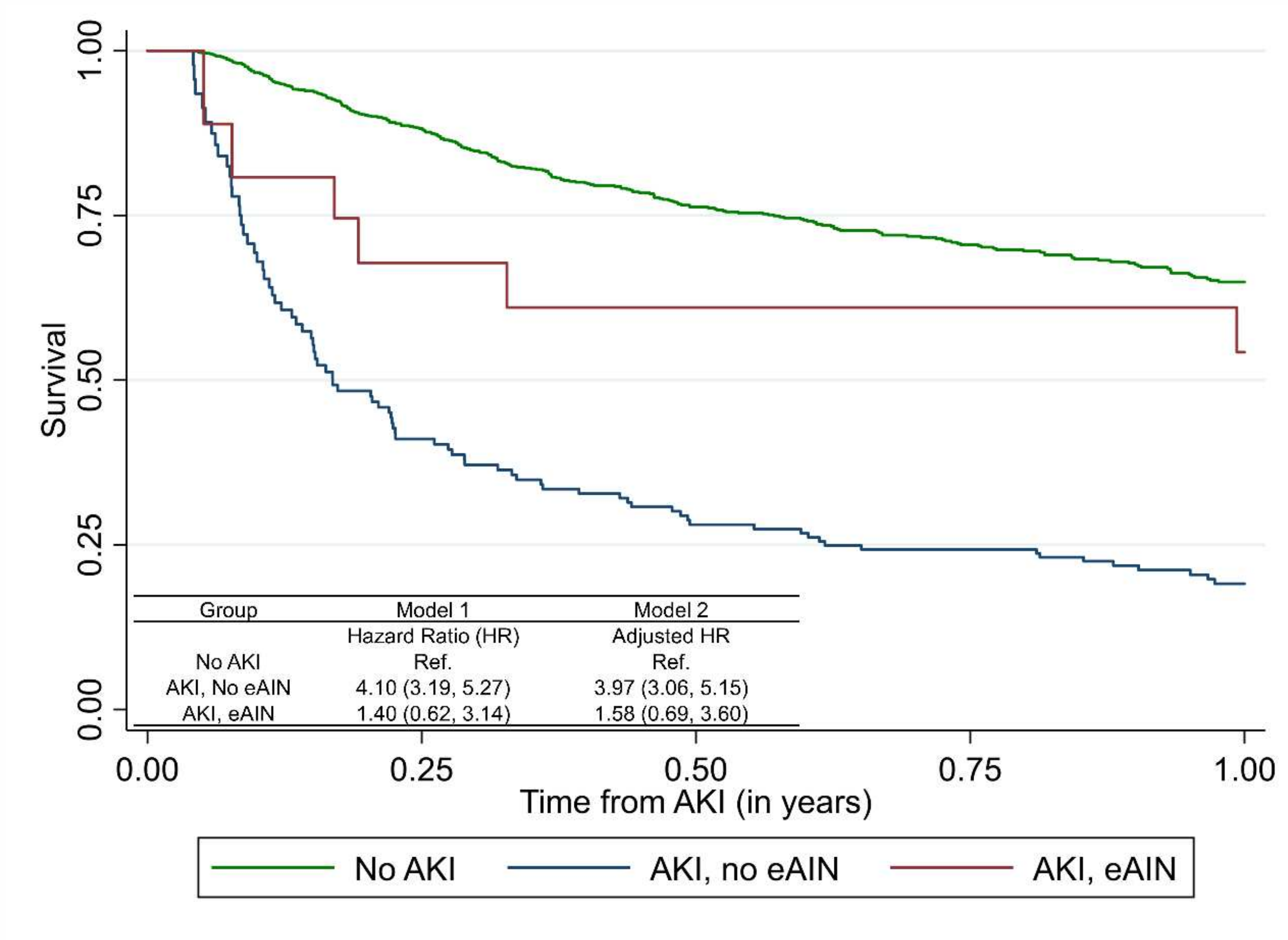

- *Association of estimated AIN with mortality in all participants initiated on immune-checkpoint inhibitor therapy using time-varying Cox proportional hazards models where exposure (presence or absence of AKI or eAIN) was treated as a time-varying covariate updated once if it occurred and patient considered as exposed for the remainder of the analysis period.*
- *Excludes those who received ICI therapy as part of a clinical trial (n=781, 34%)*
- *Follow-up starts 15 days after initiation of immune-checkpoint inhibitor therapy*
- *eAIN defined as those in the top 10% of AIN probability as determined by the diagnostic model.*
- *Model 1 tests univariable association of AKI with mortality; Model 2 controls for age, sex, race, ethnicity, presence of comorbidities (CKD, CHF, COPD, cirrhosis, diabetes, Elixhauser comorbidity score), cancer type (lung, melanoma, other), metastasis, and time-updated administration of ICI.*
- *Extended Kaplan Meier curve accounting for time-varying covariate.*
